# Agent-based modeling and bifurcation analysis reveal mechanisms of macrophage polarization and phenotype pattern distribution

**DOI:** 10.1038/s41598-019-48865-z

**Published:** 2019-09-04

**Authors:** Niloofar Nickaeen, Jafar Ghaisari, Monika Heiner, Shiva Moein, Yousof Gheisari

**Affiliations:** 1 Department of Electrical and Computer Engineering, Isfahan University of Technology, 84156-83111 Isfahan, Iran; 2 Computer Science Department, Brandenburg University of Technology, 03013 Cottbus, Germany; 3 Regenerative Medicine Research Center, Isfahan University of Medical Sciences, Isfahan, 81746-73461 Iran

**Keywords:** Multistability, Nonlinear dynamics, Population dynamics, Cytokines, Dynamical systems

## Abstract

Macrophages play a key role in tissue regeneration by polarizing to different destinies and generating various phenotypes. Recognizing the underlying mechanisms is critical in designing therapeutic procedures targeting macrophage fate determination. Here, to investigate the macrophage polarization, a nonlinear mathematical model is proposed in which the effect of IL4, IFNγ and LPS, as external stimuli, on STAT1, STAT6, and NFκB is studied using bifurcation analysis. The existence of saddle-node bifurcations in these internal key regulators allows different combinations of steady state levels which are attributable to different fates. Therefore, we propose dynamic bifurcation as a crucial built-in mechanism of macrophage polarization. Next, in order to investigate the polarization of a population of macrophages, bifurcation analysis is employed aligned with agent-based approach and a two-layer model is proposed in which the information from single cells is exploited to model the behavior in tissue level. Also, in this model, a partial differential equation describes the diffusion of secreted cytokines in the medium. Finally, the model was validated against a set of experimental data. Taken together, we have here developed a cell and tissue level model of macrophage polarization behavior which can be used for designing therapeutic interventions.

## Introduction

Complex chronic diseases are a challenge of current medicine. The insufficiency of current therapies is at least partly due to our inadequate knowledge of the mechanisms of tissue damage and regeneration. Although in tissue injuries a variety of cell types interact in a complex network, recent evidence suggests that macrophages play a critical role^1,2^. Macrophages are traditionally categorized as pro-inflammatory M1 and anti-inflammatory M2 types. However, it is now believed that they can undergo a spectrum of fates depending on their surrounding milieu and internal signaling molecules. The fate spectrum includes M1 and M2 phenotypes on the opposite ends and M2-like macrophages including M2a, M2b, M2c and mixed type phenotypes in between. Macrophage fate decision can critically affect the destiny of tissue damage. Therefore, modulating macrophage polarization seems a promising therapeutic approach to enhance intrinsic tissue regeneration potential. To specify how to manipulate the polarization process, switching gene regulatory circuits in macrophage dynamics as well as interactive communications between a macrophage and its neighboring population should be taken into account. Therefore, it is essential to develop a quantitative and predictive mathematical model to analyze these complex processes. Macrophage fate switch as a result of changes in environmental cytokine concentrations intrigued the idea that dynamic bifurcation occurs in macrophage dynamics. In the present research, bifurcation analysis properly reveals the effects of variable environmental cytokines including IL4, IFNγ and LPS on macrophage polarization and fate switches. Furthermore, mutual interactions between a macrophage and its neighboring population also affect macrophage fate through cytokine production. Therefore, such a model should consider the continuous cell population communications. An agent-based modeling scheme accounts for these features properly to model collective patterns of macrophage phenotypes which are important in disease regression and progression^3^.

Numerous experimental studies have attributed imbalance in macrophage phenotype populations to clinical presentations^4^ including neurodegenerative disorders^5^ and obesity^6^. Balance in phenotype population has been also analyzed computationally in a case of left ventricular remodeling following myocardial infarction^7,8^ by Ordinary Differential Equation (ODE) based models. To study the phenotype balance in an agent-based context, besides dynamic modeling of individual macrophages, their mutual interactions and fate determination mechanisms should also be taken into account. Although the response of macrophages to bacteria and the interactions of these cells with other immune system elements have been previously modeled, macrophage fate decision and dynamic bifurcation were ignored so far^9^. Pertsovskaya *et al*. employ bifurcation analysis to study macrophage dynamics. However, this model is focused on a single signaling pathway and does not consider the interactions of other key players^10^. Moreover, bifurcation analysis was not yet performed in an agent-based context to obtain a whole picture of macrophage pattern formation.

**Figure 1:**
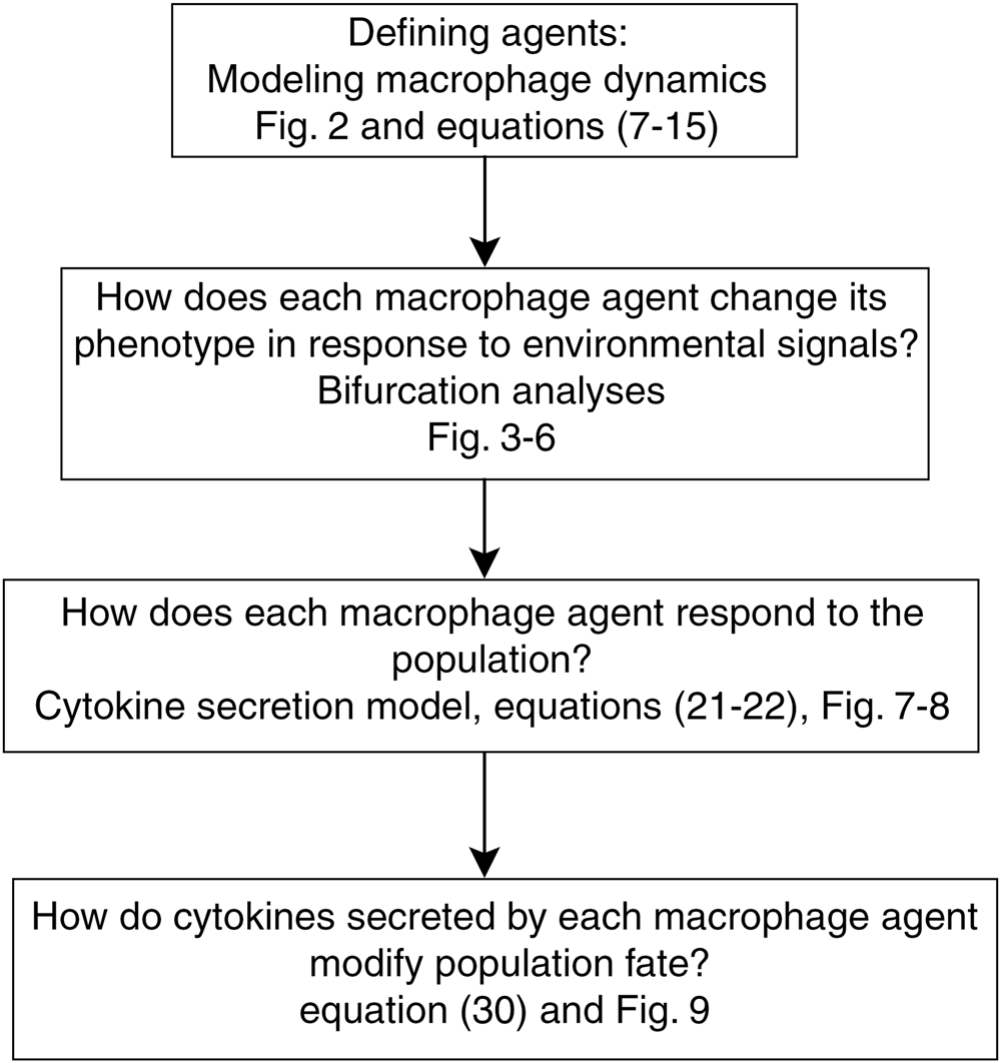
The modeling and analyses work flow.

In this research, we propose a nonlinear model of macrophages which considers dynamic bifurcation in an agent-based context. Using bifurcation analyses, the proposed model predicts macrophage polarization after exposure to different combinations of cytokines at various concentrations, which is then used to describe population phenotype patterns (Fig. 1). The results reveal new findings including that the steady states of proteins which activate molecular pathways accept specific stable activity levels with respect to each other. Relative activity levels are then identified for STAT6, STAT1 and NFκB pathways. Moreover, mathematical analysis and experimental data show that various combinations of steady states of activator proteins result in different macrophage fates and each cell changes its phenotype along the critical points of dynamic bifurcations created in response to environmental changes. Cytokine production by each phenotype is then quantitatively predicted to identify cell interactions at the tissue level based on the proposed agent-based approach. The developed two-layer model, which is constructed based on ODEs, Partial Differential Equations (PDEs) and agent-based modeling scheme, was successfully validated against experimental data from literature.

## Results

### An ODE-based model describing the dynamism of macrophage molecular interactions

**Figure 2:**
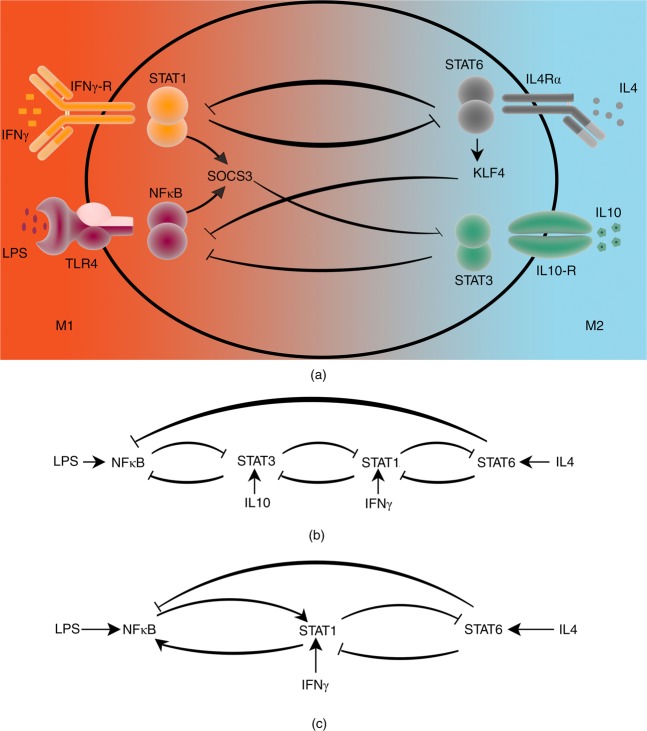
Molecular interaction graphs illustrating pathways that govern macrophage polarization. Two different types of arcs represent activator and inhibitory reactions. (**a**) Environmental signals are mediated through Toll-Like Receptor 4 (TLR4), IFNγ-R, ILR4α, and IL10-R and activate NFκB, STAT1, STAT6, and STAT3 pathways, respectively. The macrophage fate spectrum is color-coded with M1 (red) and M2 (blue) at opposite ends. (**b**) Macrophage molecular key cascades obtained by eliminating mediator proteins. (**c**) Final molecular interaction graph obtained by replacing two-cascaded double negative feedback loops between STAT1, STAT3 and NFκB by a double positive feedback loop between STAT1 and NFκB.

In order to model the macrophage dynamics, molecular interactions of macrophages were harvested from the literature. Sica *et al*. present a relatively detailed interaction map of signaling molecules and transcription factors in macrophage^5^ which is used here as a starting point for the modeling process. LPS and IFNγ signals are relayed via NFκB and STAT1, respectively, which result in SOCS3 activation. The anti-inflammatory IL10 or IL4 cytokines activate STAT3 and STAT6, respectively. These elements are connected through cascades of double negative feedback loops (Fig. 2a,b).

Redundant mediators stimulate macrophages towards specific fates. Pro-M1 mediators include TNFα, LPS and IFNγ and pro-M2 cytokines are IL4, IL13, IL10, IL6, among which IFNγ and IL4 are the main representatives, respectively^11–13^. Moreover, both IL4 and IL10 mediate augmentation of M2 polarization through STAT6 and STAT3 in a cooperative manner^14,15^. Therefore, due to the redundancy of the functions, IL4 mediated STAT6 is representatively kept in the model (Fig. 2c).

The elimination of IL10 and STAT3 makes downstream analyses more feasible without losing the key dynamics^16^. The model is simplified by replacing two cascaded double-negative feedback loops with one double-positive feedback loop. It has been shown that the input-output behavior of the aforementioned loops as well as their bifurcating behaviors are similar^17^.

In this study, the kinetic laws of the constructed ODE model follow the Wilson-Cowen structure equations^18^:

1 $$\frac{d{x}_{i}}{dt}={\gamma }_{i}(F(v)-{x}_{i}),$$

2 $${w}_{i}={w}_{i0}+\mathop{\sum }\limits_{j=1}^{N}\,{w}_{ij}{x}_{j},$$

3 $$F(\sigma w)=\frac{1}{1+{e}^{-w\sigma }}.$$

here, *x*_*i*_ is the concentration of the ith protein, *F* is a function according to equation (3), *γ*_*i*_ tunes the production rate of the ith protein, and *w*_*i*_ determined by equation (2) represents the net effect on protein *i*. The factor *w*_*ij*_ which is the effect of the j^th^ protein on the i^th^ one is positive if the protein *j* activates the protein *i* and negative if the protein *j* inhibits the protein *i*. *w*_*i*0_ represents the initial rate of change for the ith protein and *σ* determines the steepness of *F*(*σw*) versus *w*. Finally, *N* is the number of proteins or genes in the network.

With some mathematical manipulations, Tyson *et al*. transformed the Wilson-Cowen structure equations into^18^:

4 $$\frac{d{x}_{i}}{dt}={\gamma }_{i}\frac{{A}_{i}(1-{x}_{i})-{B}_{i}{x}_{i}}{{A}_{i}+{B}_{i}},$$

5 $${A}_{i}={\exp }({\sigma }_{i}({a}_{i0}+\mathop{\sum }\limits_{j=1}^{N}{a}_{ij}{x}_{j})),$$

6 $${B}_{i}={\exp }({\sigma }_{i}({b}_{i0}+\mathop{\sum }\limits_{j=1}^{N}\,{b}_{ij}{x}_{j})).$$

here, *A*_*i*_ and *B*_*i*_ are the exponential activation and inhibition rates of the i^th^ protein, respectively. *a*_*ij*_ and *b*_*ij*_ represent the activation and inhibition of the i^th^ protein by the j^th^ protein and can vary in a continuum interval between 0 and 1. The initial rate of change for the protein *i* is defined using parameters *a*_*i*0_ ≤ 0 and *b*_*i*0_ ≤ 0. Based on equations (4–6), a system of ODEs was constructed to model macrophage molecular interactions (equations 7–15). In all equations, it has been assumed that ∀i:*σ*_*i*_ = 8, *γ*_*i*_ = 1.

7 $$\frac{d(NF\kappa B)}{dt}=\frac{{A}_{1}(1-NF\kappa B)-{B}_{1}(NF\kappa B)}{{A}_{1}+{B}_{1}},$$

8 $${A}_{1}=exp({\sigma }_{1}(\,-\,1.5+LPS+STAT1)),$$

9 $${B}_{1}=exp({\sigma }_{1}(-0.1+STAT6)).$$

10 $$\frac{d(STAT1)}{dt}=\frac{{A}_{2}(1-STAT1)-{B}_{2}(STAT1)}{{A}_{2}+{B}_{2}},$$

11 $${A}_{2}=exp({\sigma }_{2}(\,-\,0.5+IFN\gamma +NF\kappa B)),$$

12 $${B}_{2}=exp({\sigma }_{2}(\,-\,0.5+STAT6)).$$

13 $$\frac{d(STAT6)}{dt}=\frac{{A}_{3}(1-STAT6)-{B}_{3}(STAT6)}{{A}_{3}+{B}_{3}},$$

14 $${A}_{3}={\exp }({\sigma }_{3}(IL4)),$$

15 $${B}_{3}=exp({\sigma }_{3}(\,-\,0.05+STAT1)).$$

The parameters *a*_*ij*_ and *b*_*ij*_ were considered as “1”, if there is an arc in the interaction graph, and “0” otherwise. The initial change rates *a*_*i*0_ and *b*_*i*0_ are all confined to the ranges of −1.5 ≤ *a*_*i*0_ ≤ 0 and −0.5 ≤ *b*_*i*0_ ≤ 0 and are, along with *γ*_*i*_ and *σ*_*i*_, inspired by the study of Tyson *et al*.^18^. These parameters are chosen so that to stimulate critical dynamic behaviors of the model.

### Bifurcation analysis reveals switches in macrophage phenotype selection

Macrophages are able to perform a variety of divergent functions in response to environmental stimuli without changing their internal structure of molecular interactions^4,5,14,19,20^. Hence, dynamic bifurcation analysis, known as analyzing qualitative changes in system behavior due to alteration of system parameters, seems a suitable method for the analysis of macrophage phenotype selection. Assume

16 $$\frac{d\underline{x}}{dt}=f(\underline{x},\underline{\mu }).\,\underline{x}\in {R}^{n},\,\underline{\mu }\in {R}^{m}$$

here, $\underline{x}$ is the state vector and $\underline{\mu }$ is the parameter vector of the system. Let *μ* be a scalar and the equilibrium point of the system be $(\underline{\bar{x}},\mu )=(\underline{{{\rm{x}}}_{0}},{\mu }_{0})$. $\underline{\bar{x}}$ is the steady state of the state vector $\underline{x}$. A local dynamic bifurcation occurs at *μ* = *μ*_0_, if the system flow near the equilibrium point $\underline{{{\rm{x}}}_{0}}$ is not qualitatively the same for *μ* = *μ*_0_ and any *μ* near *μ*_0_. $(\underline{\bar{x}},\mu )=(\underline{{{\rm{x}}}_{0}},{\mu }_{0})$ is called bifurcation point^21^. In this paper, bifurcation analyses were all performed numerically using the XPP AUTO software^22^.

**Figure 3:**
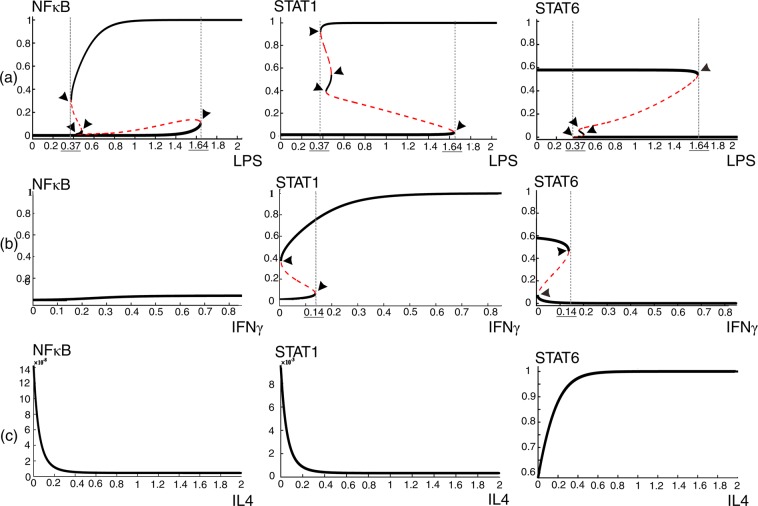
Bifurcation analyses of equations (7–15) for one parameter. Bifurcation diagrams of mediator proteins NFκB, STAT1 and STAT6 considering (**a**) LPS, (**b**) IFNγ, (**c**) IL4 as bifurcation parameter. Solid lines and dashed lines represent stable and unstable steady states, respectively. Arrow heads point to saddle-node bifurcation points and critical switching borders are specified by dotted lines and underlined values.

To perform local bifurcation analysis on the proposed ODE model (equations 7–15), LPS, IL4, and IFNγ were assumed as variable parameters and were varied in particular ranges. The steady states of STAT6, STAT1 and NFκB in response to various concentrations of LPS, IFNγ and IL4 were determined as a result. At the first step, bifurcation analysis was performed by considering each of these signaling molecules as the bifurcation parameter, while ignoring the other two. Varying the concentrations of LPS, three stable and two unstable steady states were observed for each STAT6, STAT1 and NFκB molecule (Fig. 3a). The points in which stable and unstable equilibriums collide and annihilate each other, known as saddle node bifurcation points, are specified with arrow heads. For low and high concentrations of LPS, *LPS* ≤ 0.37 and *LPS* ≥ 1.64 respectively, each of the three mediators were observed at a single stable level. But at medium concentrations 0.37 ≤ *LPS* ≤ 1.64, there are more than one choice of stable states allowing a switch-like performance. The critical values of LPS, in which the dynamic behavior of the system changes, are indicated with dotted lines. By increasing or decreasing LPS concentration and crossing the critical values, STAT1, STAT6 and NFκB change their steady states in a switch like manner. These switches allow the system to take on new operating modes by changing steady state activity levels of its pathways. As each of these pathways mediates specific downstream expressions, different fates are expected for a macrophage in different operating modes. Similarly, when IFNγ is considered as the variable parameter in the absence of LPS and IL4, saddle node bifurcations are observed for STAT1 and STAT6 (Fig. 3b). No bifurcation was observed in the presence of various concentrations of IL4 alone (Fig. 3c).

**Figure 4:**
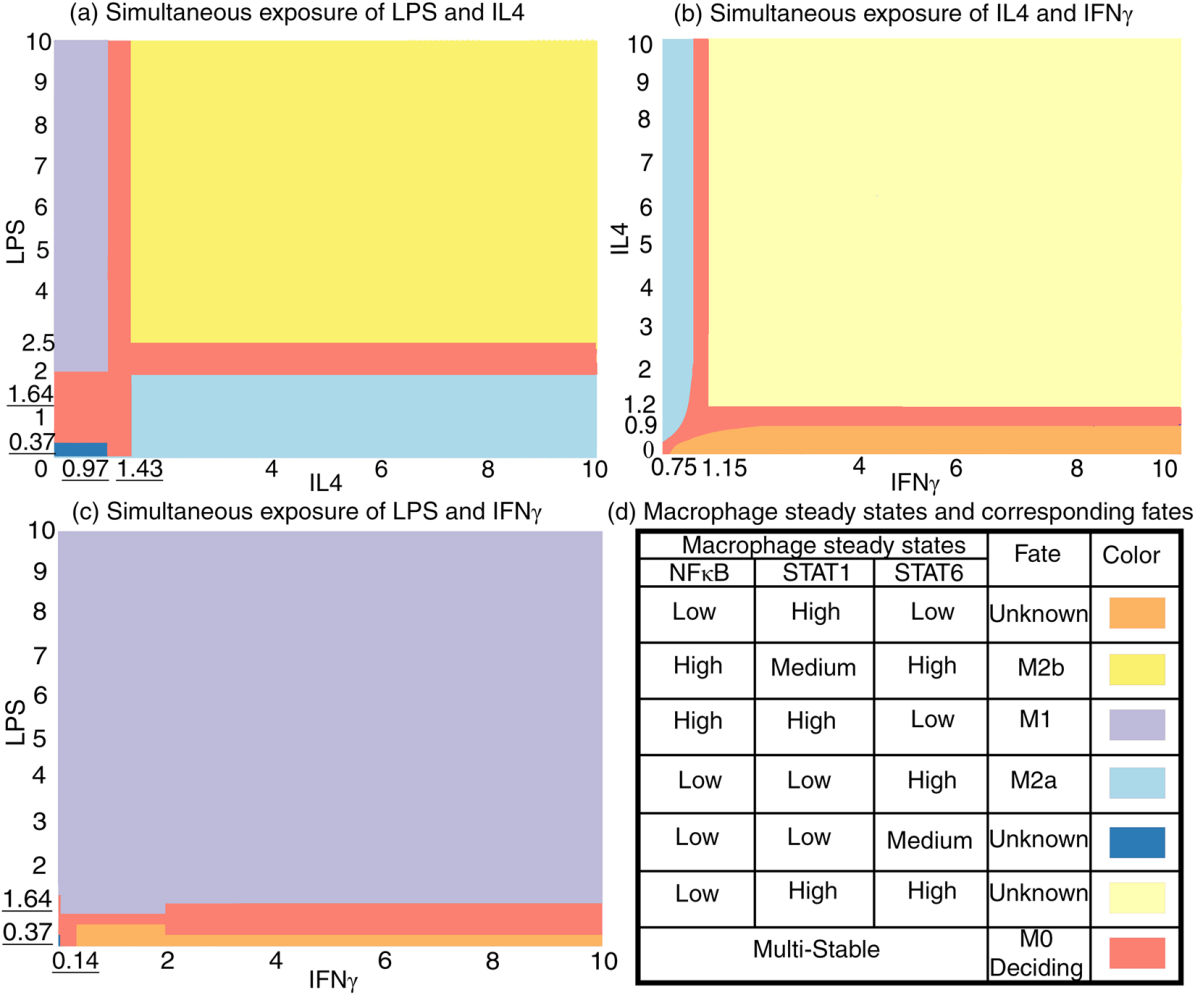
Bifurcation analyses of equations (7–15) for two parameters. Simultaneous exposure of macrophage to different concentrations of (**a**) LPS and IL4, (**b**) IL4 and IFNγ, (**c**) LPS and IFNγ results in (**d**) different steady state profiles for the mediator proteins “NFκB, STAT1, STAT6” which determine macrophage phenotype. Underlined values are critical borders obtained from one-parameter bifurcation analysis.

To further proceed with macrophage phenotype analysis, the effect of the simultaneous exposure of dual combinations of LPS, IFNγ, and IL4 was assessed using the critical values obtained from the previous step. For instance, NFκB, STAT1, and STAT6 steady states were numerically calculated when LPS varies along the critical values of IL4, and vice versa (Fig. 4a). Similarly, the steady states of these three mediators were investigated in the presence of IFNγ and IL4 (Fig. 4b), or IFNγ and LPS (Fig. 4c). In response to various concentrations of the stimulating molecules, based on the stable levels of NFκB, STAT1, and STAT6, different regions could be identified. Interestingly, the boundaries of these regions are areas in which saddle-node bifurcation and multi-stability occurs and macrophage chooses between different stable steady states. In these multi-stable conditions, saddle-node bifurcation points may be lost or produced due to the alteration of the corresponding parameters.

As fate-determining signaling cascades in macrophage are activated through one of the NFκB, STAT1 and STAT6 proteins^5^, stable steady states of these proteins determine the activity levels of their corresponding pathways. Indeed, the NFκB, STAT1, and STAT6 concentrations represent the macrophage state in response to environmental stimuli. Therefore, we inferred the macrophage phenotype from this triplet profile (Fig. 4d). For instance, in high concentrations of either LPS alone (Fig. 3a) or the combination of LPS and IFNγ (Fig. 4c), the triplet “NFκB, STAT1, STAT6” is at “High, High, Low” levels, which, based on previous knowledge^4,23^, we consider as M1 phenotype. Similarly, in high concentrations of IL4 (Fig. 3c), the profile is “Low, Low, High” that can be attributed to M2a^4^. In addition, in high concentrations of LPS and IL4 (Fig. 4a), the profile is “High, Medium, High” corresponding to M2b^4^. However, we appreciate that the biological significance of some profiles remains unknown.

**Figure 5:**
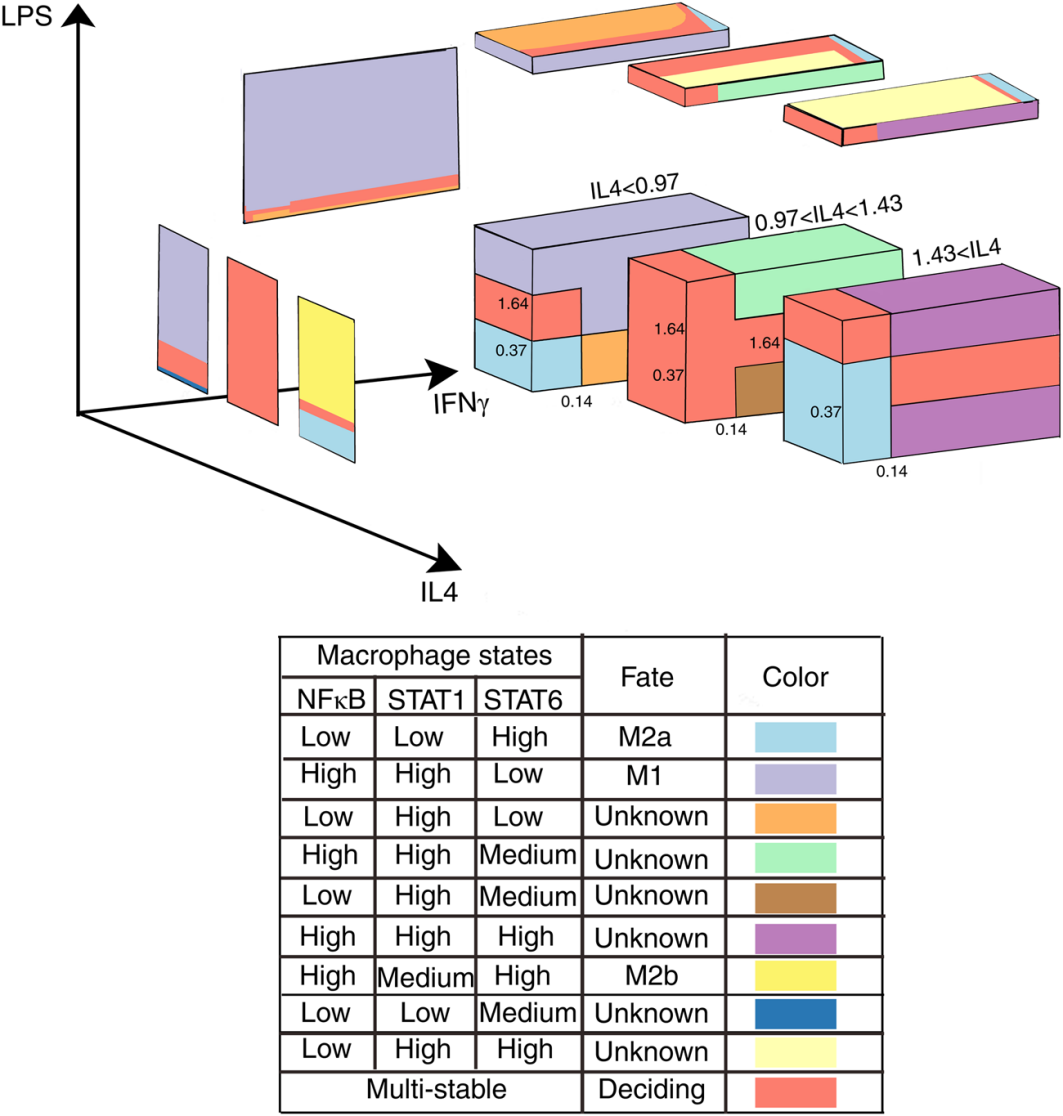
Bifurcation analyses of equations (7–15) for three parameters. Simultaneous exposure of macrophage to different concentrations of LPS, IL4 and IFNγ result in different steady state profiles for “NFκB, STAT1, STAT6” and accordingly into different phenotypes, compare Fig. 6 for phenotype reachability. The left, up and back panels which depict macrophage states in LPS-IL4, IL4-IFNγ and LPS-IFNγ are illustrated separately, compare Fig. 4.

**Figure 6:**
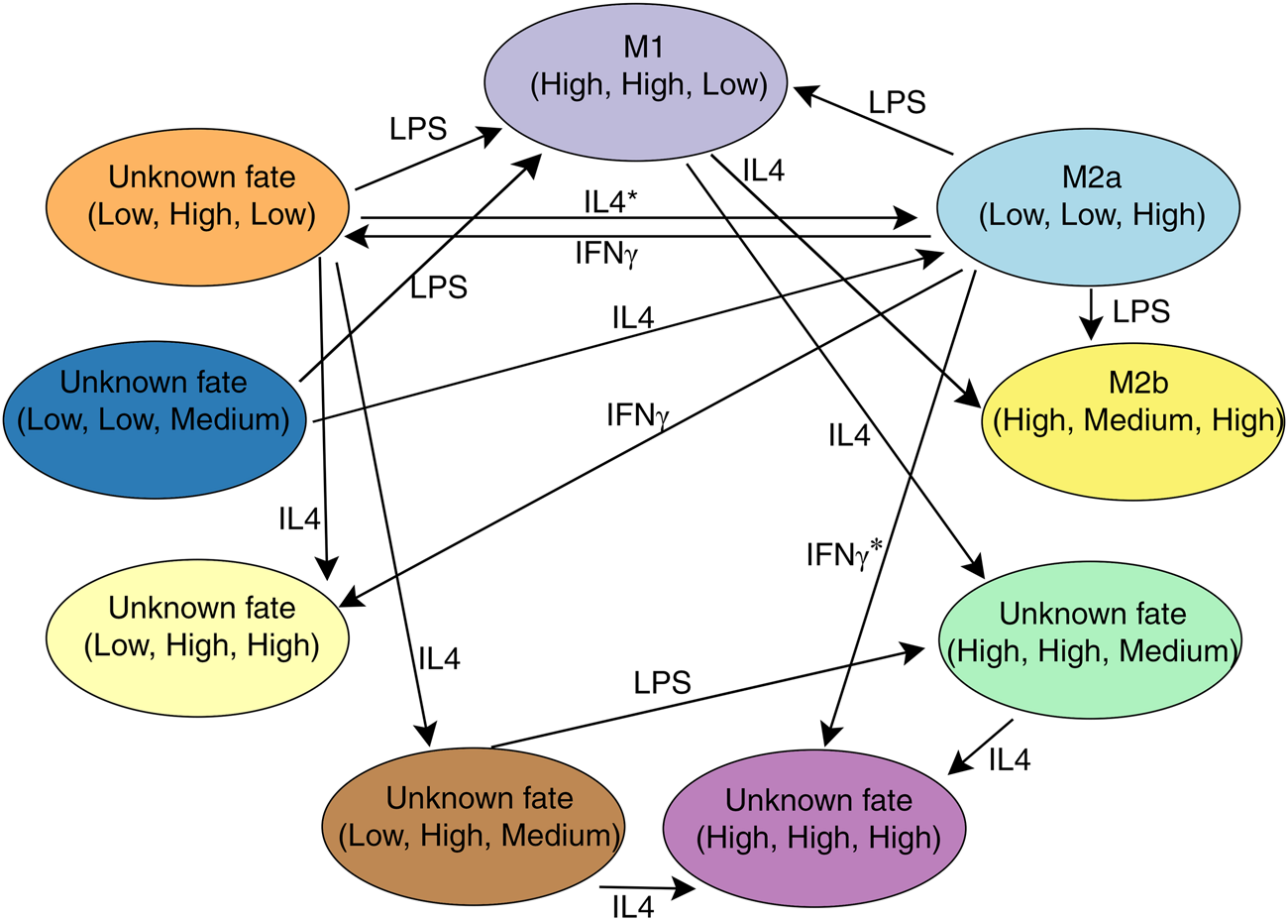
Reachability for the nine specified phenotypes as determined by bifurcation analysis, compare Figs 3–5. Each arc is labeled with the name of the corresponding cytokine causing the fate change. The arc labeled with IL4* switches the macrophage phenotype only, if the IFNγ concentration is low. The arc labeled with IFNγ* switches the macrophage phenotype only, if the LPS concentration is low.

Using our model, compare equations (7–15), we managed to predict the macrophage phenotype based on the steady states of its internal mediators in the simultaneous presence of all three stimulating molecules. Although a maximum amount of 27 combinations of high, medium, and low levels of the three internal mediators are possible, the gene regulatory circuits of macrophages allow only 9 phenotypes (Fig. 5 and also see Supplementary Table 1), probably because they are beneficial and hence evolutionary conserved. These phenotypes are able to change their fates based on proper stimuli (Figs 4 and 5). Reachable fates along with their corresponding stimuli are depicted in (Fig. 6). Our results are in accordance with current experimental knowledge indicating that IL4 cytokine skews macrophage to M2-like phenotypes, while LPS skews macrophage to the M1 phenotype. The results also agree with current popular spectrum-like models of macrophage fate. The other connections remain to be evaluated experimentally.

In summary, macrophage fate decision in response to LPS, IFNγ, and IL4 can be simulated using the ODE model of macrophage signaling reactions and bifurcation analysis.

### An agent-based scheme interacting with ODE-based intracellular model to describe macrophage population behavior

Macrophage fate determination is influenced by external stimuli including the cytokines present in the surrounding micro environment (Figs 3–6). In turn, each macrophage secrets special cytokines based on its phenotype which affects the neighboring cells (equations (21–22, 30)). Therefore, each macrophage is permanently in a dynamic mutual interaction with the macrophage population. Indeed, macrophage population polarization seems to be controlled by a decentralized control algorithm and so an agent-based modeling approach is used to simulate the behavior of a population of macrophages. This tissue-level model constantly exchanges data with the constructed cell-level ODE model.

Bifurcation analysis described in the previous section allows identifying cell polarization based on the external signaling molecules. However, to determine the mutual interactions of each macrophage with the population, it is also critical to model the cytokines secreted by each phenotype as well as the diffusion of secreted cytokines in the nearby environment. These issues are addressed in the following sections.

**Figure 7:**
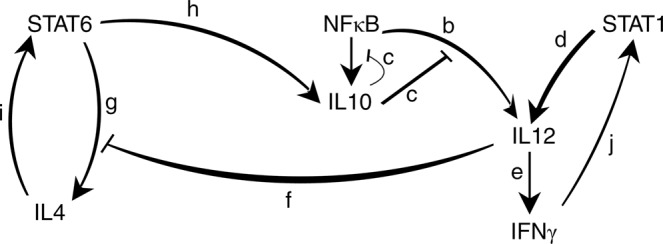
IL4 and IFNγ production in macrophage.This molecular interaction graph illustrates reactions involved when macrophage responds to the population by producing cytokines. The graph depicts reactions governing IL4 and IFNγ cytokine production by the NFκB, STAT1 and STAT6 pathways. IL10 and IL12 are mediator proteins relaying cytokine production. The graph is composed of two types of inhibitory and activator arcs and arc labels specify reaction rate parameters. Compare Fig. 2 which illustrates macrophage molecular pathways involved when macrophage is stimulated by cytokines from population.

To define the cytokines secreted by each macrophage in response to activation of STAT6, STAT1 or NFκB, a molecular interaction graph is constructed according to previous experimental studies (Fig. 7). Briefly, NFκB and STAT1 induce the production of IL12, which skew the cell to M1 phenotype both through up-regulation of IFNγ and the inhibition of IL4 production^5,24,25^. STAT6 deviates towards the other end of the phenotype spectrum through production of IL4 in a positive feedback loop. STAT6 also induces IL10, which inhibits IL12 production^25,26^. Notably, in addition to the strong effect of NFκB on M1 activation, it can induce M2 through IL10. However, this effect is trivial due to the IL10 auto-negative feedback which hinders its M2 promoting effect^25^.

Based on this interaction graph, a set of ODEs is proposed to describe macrophage cytokine production using Mass Action kinetics. Inhibitory reactions are modeled by subtracting the product of inhibition rate and inhibitor concentration from the corresponding rate terms.

17 $$\frac{d(IFN\gamma )}{dt}=e(IL12)-j(IFN\gamma ),$$

18 $$\frac{d(IL12)}{dt}=d(STAT1)+b(NF\kappa B)-c(IL10)-f(IL12)-e(IL12),$$

19 $$\frac{d(IL10)}{dt}=a(NF\kappa B)-2c(IL10)+h(STAT6),$$

20 $$\frac{d(IL4)}{dt}=g(STAT6)-i(IL4)-f(IL12).$$

As the time scale of intra-cellular molecular reactions is much smaller than that of inter-cellular communications^27^, the internal reactants are considered in their steady states (equations (17–20)). According to this assumption and using the proposed ODE model, the steady state concentrations of IFNγ and IL4 produced in response to STAT1, STAT6, and NFκB are calculated as below. The bar mark indicates the steady state concentration of reactants.

21 $$\overline{IL4}=(\frac{g}{i}+\frac{fh}{2i(e+f)})\,\bar{STAT6}-\frac{fd}{i(e+f)}\,\bar{STAT1}-\frac{f(2b-a)}{2i(e+f)}\,\bar{NF\kappa B}\,{\rm{I}}{\rm{f}}\,\bar{{\rm{I}}{\rm{L}}4} > 0,\,else\,0.$$

22 $$\bar{IFN\gamma }=\frac{ed}{(e+f)j}\bar{STAT1}+\frac{e(2b-a)}{2(e+f)j}\bar{NF\kappa B}-\frac{eh}{2(e+f)j}\,\bar{STAT6}\,{\rm{I}}{\rm{f}}\,\bar{IFN\gamma } > 0,\,else\,0.$$

In order to solve the above equations, the constant rates as well as the steady state concentrations of STAT6, STAT1 or NFκB need to be identified. The rates of all reactions are harvested from the literature (Table 1), excluding parameter “*h*”. To estimate the parameter “*h*”, we used the fact that when a macrophage is exposed to high levels of IFNγ and IL4 simultaneously, it displays a (M1/M2) mixed type and secretes both IL4 and IFNγ as response. Thus, in this specific environmental condition, it is expected that macrophage produces both IFNγ and IL4 simultaneously, i.e.

23 $$\overline{IL4} > 0.$$

24 $$\overline{IFN\gamma } > 0.$$

when exposed to high levels of IFNγ and IL4, the triple (NFκB, STAT1, STAT6) corresponds to the (Low, High, High) mode. By assuming Low concentration of $\overline{NF\kappa B}$ zero based on the plots in Fig. 3 and substituting the parameter values of Table 1, equations (23) and (24) are simplified to (25) and (26) as a function of the parameter *h*.

25 $$\overline{IL4}=(2+809.09h)\,\overline{STAT6}-0.22\,\overline{STAT1} > 0.$$

26 $$\overline{IFN\gamma }=0.009\,\overline{STAT1}\,-\,(32.35h)\,\overline{STAT6} > 0.$$

**Table 1:** The constant rates of the reactions involved in cytokine production by macrophages in response to the activation of STAT6, STAT1 or NFκB.

Parameter	Rate	Reference
a	4.15 × 10^−4^	^52^
b	4.15 × 10^−4^	^52,53^ *
c	1.587 × 10^−4^	^53,54^ *
d	1.38 × 10^−4^	^53^
e	1.157 × 10^−5^	^55^
f	9.26 × 10^−5^	^56^
g	1.1 × 10^−3^	^57^
h	2 × 10^−3^	Estimated
i	5.5 × 10^−4^	^58^
j	1.7 × 10^−3^	^59^

^*^Reaction rates for parameters with multiple suggested values are estimated by eliminating outliers with different time scales relative to the rest and then averaging the remaining parameters.

As for $\overline{STAT1}$ and $\overline{STAT6}$, normal protein abundances of STAT1 and STAT6 are used (Table 2). By substituting protein abundances in (25) and (26) and solving the resultant inequalities, *h* is calculated by equation (27).

27 $$0.001 < h < 0.0036$$

The parameter *h* is assumed to be 0.0020 for further simulations.

In bifurcation analyses, we have subjectively classified STAT1, STAT6 and NFκB concentrations into Low, Medium, and High. To have a rough estimate, the average natural concentrations of these proteins according to PaxDb^28^ were considered as Medium level. High and Low concentrations were assumed as twice of the normal abundance or zero, respectively. The NFκB family has different members, among which RELA and NFκB1 are active in macrophage signaling^5^. The mean concentration of RELA is lower than that of NFκB1 and hence is considered as the normal abundance of the NFκB protein complex as it can be a rate limiting factor (Table 2).

**Table 2:** Normal protein abundances harvested from the PaxDb database for molecules involved in the macrophage polarization process.

Protein	Abundance (ppm)
STAT1	531
STAT6	41.3
NFκB	
NFκB1 (P50)	35.3
RELA (P65)	26.5
RELB	1.34
REL	24.9

In summary, based on the above equations and assumptions, the cytokines secreted by each macrophage can be quantitatively modeled. However, to precisely describe the effect of each macrophage on the population, the diffusion rates of the cytokines as well as cell movements need to be considered, too.

To model cytokine diffusion in the region where the population resides, a reaction-diffusion system is defined by the following Partial Differential Equation (PDE):

28 $$\frac{\partial \varphi (t,r)}{\partial t}=\nabla .(D(\varphi ,r)\times \nabla \varphi (r,t))+f(\varphi ,r,t).$$

here, *φ*(*t*, *r*) is the cytokine concentration which is a function of the position *r* and time *t*. *f*(*φ*, *r*, *t*) is a source term that specifies macrophage cytokine production in space *r* and is defined by equation (21) and equation (22). *D* is the diffusion coefficient, which for large molecules, such as most of the proteins, can be approximated with the following equation^29^:

29 $$D=\frac{kT}{6\pi \mu R}.$$

here, *R* is the radius of the protein which is assumed to be a sphere, *k* is the Boltzmann’s constant, *μ* is the solvent viscosity, and *T* is temperature in Kelvin. Therefore, *D* can be assumed to be constant and equation (28) is simplified as follows:

30 $$\frac{\partial \varphi (t,r)}{\partial t}=D({\varphi }_{xx}+{\varphi }_{yy})+f(\varphi ,r,t).\,$$

Based on previous experiments^29–31^, D is estimated as 1800 $\frac{micro{n}^{2}}{min}$ for both IL4 and IFNγ cytokines. For simulation purposes, the inflammation site is assumed as a circular area in which macrophages reside around point (0, 0) with a normal distribution and a variance of *σ*^2^ = 0.25. The Dirichlet boundary condition is set to zero on a circular boundary of radius 4. To allow for cell movements, each macrophage is able to randomly move for a maximal distance of 2*σ*^2^ in each simulation run.

**Figure 8:**
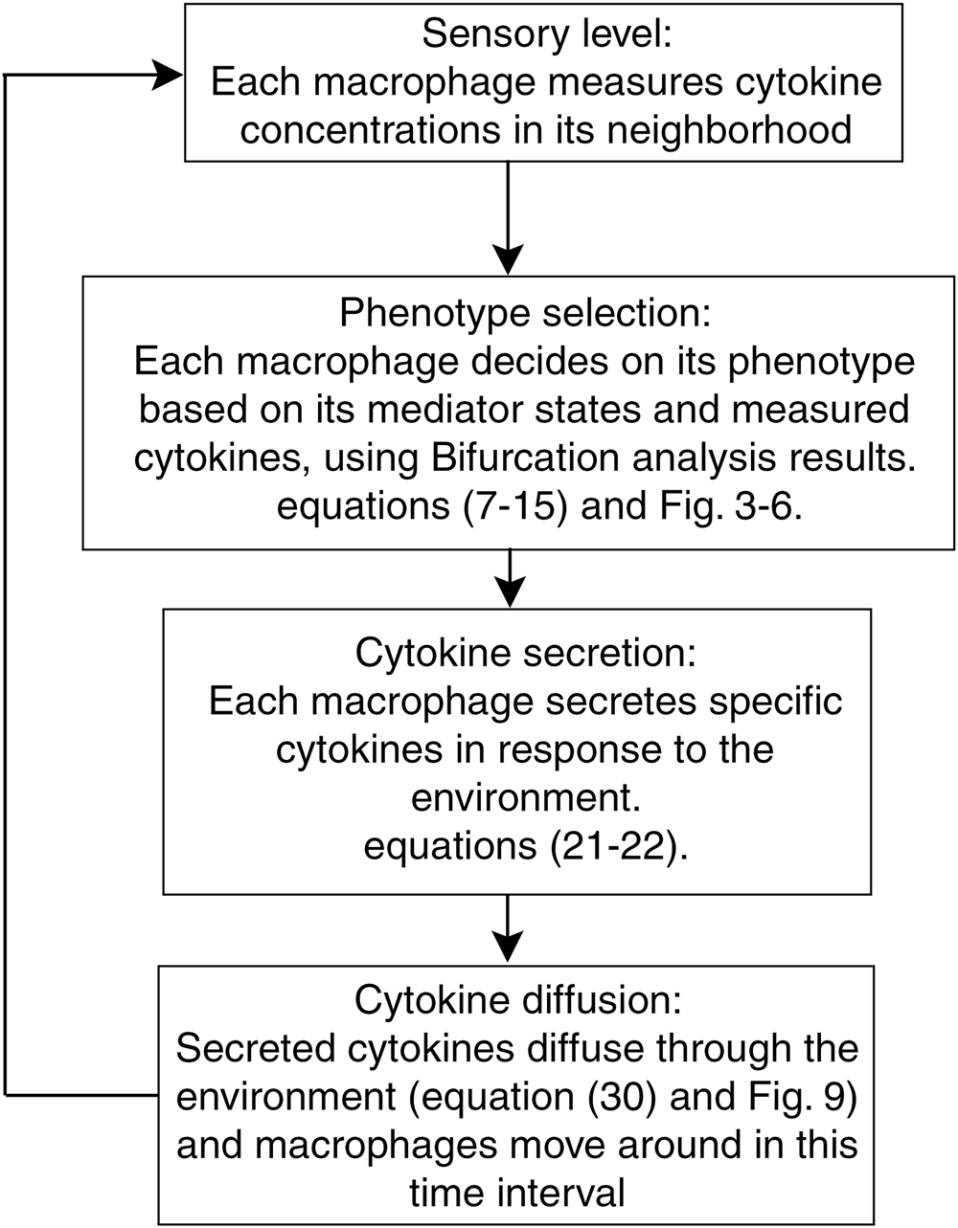
Cyclic two-level model simulation of cytokine-based interactions of macrophage population.

In summary, based on equations (7–15) and using bifurcation analysis, the expression levels of STAT1, STAT6 and NFκB in response to external stimuli are determined. Then, using the equations (21 and 22) the concentrations of cytokines secreted by each macrophage are identified. Eventually, by equation (30) the diffusion rates are considered to identify the concentration of the cytokines in time and space. Also, random cell movements are taken into account. These events are repeated every Ts = 0.1 seconds (Fig. 8) where, Ts indicates sampling time in population simulations. During time range (k Ts, (k + 1) Ts) kϵN, secreted cytokines diffuse throughout the environment and adjust cytokine concentrations. At each kTs, kϵN seconds, macrophages sense cytokine concentrations in their vicinity and decide on their fate and accordingly on their cytokine secretion. This two-layer model considers both intracellular interactions of macrophage and tissue-level communications between a single macrophage and its population. Therefore, the behavior of macrophage populations in different environmental conditions can be simulated. To validate the model, the model-based predictions are compared in the next section with experimental observations reported by previous investigators.

### Model validation

#### Case 1

Although differentiation of naïve macrophages into different phenotypes has previously been investigated thoroughly, few studies have focused on the role of intra-cellular mediating molecules for cell differentiation. Li *et al*. demonstrated by stimulating M0 macrophages with LPS/IFNγ or IL4/IL3 that they skew to M1 or M2 phenotypes, respectively^32^. In M1-differentiated cells, NFκB and STAT1 increased, compared to control experiments where STAT6 did not vary. On the other hand, in M2 macrophages, STAT6 increased, while no significant changes were detected for NFκB and STAT1. This experimental observation is in agreement with the bifurcation analyses in our computational model that in response to IL4 the concentrations of “NFκB, STAT1, STAT6” are at “Low, Low, High” levels, respectively (Fig. 3c). Also, after the simultaneous exposure to LPS and IFNγ, the profile of this triplet would be “High, High, Low” (Fig. 4c).

#### Case 2

Appropriate macrophage population phenotype shift is a crucial effect in the regeneration of salamander heart and limb^33^, zebra fish fin^34^ and mammalian organs^2^. Also, a transient M1 activation followed by substitution with M2 cells is observed in self-limiting kidney injuries, while the continuous presence of M1 macrophages is associated with progressive tissue fibrosis^35^. In spite of the well-recognized importance of this phenomenon, it is yet elusive if the fate shift at the population level is solely mediated through immigration and polarization of monocytes or if trans-differentiation of terminally differentiated macrophages do also account^36,37^. Edin *et al*. have demonstrated that both M1 and M2 macrophages can be trans-differentiated to the opposite fate upon treatment with IL4 and LPS/IFNγ, respectively^38^.

**Figure 9:**
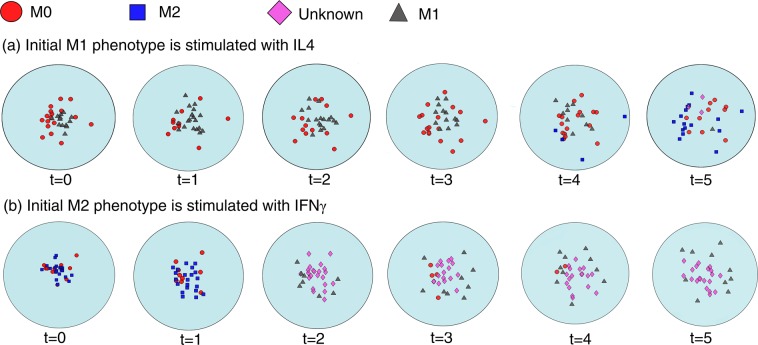
Simulation results of the PDE according to equation (30). Macrophage population dominated by (**a**) M1, (**b**) M2 phenotypes are exposed to IL4 and IFNγ cytokine, respectively. As a result, each sub-population skews to the opposite phenotype.

To reproduce this data, we started the simulation with either M1 or M2 dominant populations of thirty macrophages and exposed them to high levels of IL4 or LPS/IFNγ, respectively. The initial M1 or M2 dominant populations were created by stimulating the naïve population with a high initial concentration of M1 or M2 inducing cytokines, i.e. IFNγ and IL4, respectively, compared to the other cytokines. Each polarized population was then stimulated with the opposite cytokine stimulus. M1 dominant population was exposed to IL4 and M2 dominant population was exposed to IFNγ. The simulations were performed using the parabolic solver in MATLAB. After several time steps, each population was skewed to the opposite side (Fig. 9a,b). This finding, in agreement with the observations of Edin *et al*. indicates that terminally differentiated macrophages have high plasticity and can change their phenotype in response to signals received from the environment. Notably, it was observed that when the initial population is totally made up of one extreme of the phenotype spectrum, the other fate appears rarely even after a long time interval. However, when the initial population also consists of naïve macrophages (M0), the population skews to the opposite side more rapidly. That is because M0 macrophages easily skew in response to environmental cytokines and produce cytokines that stimulate neighboring cells to trans-differentiate.

## Discussion

Macrophage polarization governs tissue homeostasis, regeneration and remodeling by creating various phenotype patterns which are known to affect disease development or regression^3^. Thus, targeting macrophage polarization in order to maintain a balance between different phenotypes has been proposed as a therapeutic strategy^39,40^. This study aimed at developing a quantitative and predictive model of macrophage polarization that can potentially be applied to propose novel therapies.

We have generated a two-layer nonlinear mathematical model, in which internal interactions and reciprocal cell population communications are described at the cell and tissue levels, respectively. Bifurcation analysis was performed on the ODE model of gene regulatory circuits, which demonstrated switch-like behaviors allowing macrophages to choose different fates. Each of the key internal signaling mediators, namely NFκB, STAT1, and STAT6, were observed at different steady states, inducing various combinatorial profiles which were attributed to macrophage phenotypes. Nine specific profiles were recognized, three of which were attributed to known macrophage phenotypes M1, M2a and M2b based on their protein activity level and according to the literature. The six remaining phenotypes are yet to be recognized. Using the data obtained from the first layer, a PDE model was constructed to describe the secretion and diffusion of the cytokines secreted by each cell. Then, the effect of these cytokines on neighboring macrophages was assessed allowing for the simulation of cell-community interactions. The model was successfully validated against experimental data. It also reproduced repolarization of differentiated M1 and M2 macrophages in response to opposite stimuli indicating the plasticity of these cells.

Cell populations are regulated by distributed leadership instead of central control. Therefore, agent-based modeling is a rational approach to describe their behavior. Although the population could be modeled by ODEs, agent-based modeling allows following spatial and temporal phenotype patterns, compromising between details and computational cost, and adding stochasticity to individual macrophages. Tumor growth^27,41^, the geometric pattern of cell populations^42^, monocyte trafficing to tissues^43^, and wound closure^44^ are among the phenomena modeled by agent-based schemes. Furthermore, although many biological processes are continuous, some others, specifically those governing fate determination, are discrete allowing qualitative shifts in cell phenotypes. Accourdingly, bifurcation analysis has been widely utilized to study such phenomena. The formation of endocrine and exocrine lineages in pancreas^45,46^ as well as xenopus embryo development^47^ have been studied using bifurcation analysis and the underpinning gene regulatory motifs have been identified^48–50^. In addition, bifurcations in agent-based models have been assessed to simulate the collective behavior of bird flocks and bee swarms^51,52^ in response to changes in agent characteristics. However, to the best of our knowledge, the proposed approach which is based on bifurcation analysis of the ODE model of single cells and connecting it with an agent-based model of the population has not been described so far. The cell and tissue layers of the model are in a permanent mutual communication in spite of different time scales. This novel modeling strategy can potentially be applied to further the understanding of complex multi-agent biological systems and pave the way for the development of more efficient therapeutics.

## Conclusion

Macrophage behaviors are affected by changes in its parameter set as well as changes of incoming signals from neighboring population. Signaling pathways in macrophage are composed of regulatory loops which are able to switch its fate in response to different concentrations of environmental cytokines through dynamic bifurcation. New findings based on our model suggest that in response to different concentrations of environmental cytokines, the relative steady state concentration levels of activator proteins in different macrophage pathways accept specific concentration values with respect to each other. The results also suggest that this relative activity level defines the macrophage phenotype. Therefore, collective phenotype patterns in macrophage populations are justifiable by cytokine-based interactions between a single macrophage and its population. Accordingly, an agent-based model of macrophage population was proposed in which interacting rules between agents were defined by using results of a formerly performed bifurcation analysis on macrophage dynamics. The model was then validated by successfully producing anticipated phenotype patterns induced by IL4 and IFNγ cytokines. This validated model is efficient in promoting novel therapeutic approaches by simulating clinical cases and their response to candidate medications.

## Supplementary information

Supplementary Table S1

## Data Availability

The datasets generated during and/or analyzed during the current study are available from the corresponding author on reasonable request.

## References

[1] Vannella KM, Wynn TA (2017). Mechanisms of Organ Injury and Repair by Macrophages. Annual Review of Physiology.

[2] Wynn TA, Vannella KM (2016). Macrophages in tissue repair, regeneration, and fibrosis. Immunity.

[3] Sica A, Erreni M, Allavena P, Porta C (2015). Macrophage polarization in pathology. Cellular and molecular life sciences.

[4] Fraternale A, Brundu S, Magnani M (2015). Polarization and repolarization of macrophages. J Clin Cell Immunol.

[5] Sica A, Mantovani A (2012). Macrophage plasticity and polarization: *in vivo* veritas. The Journal of clinical investigation.

[6] Shapouri‐Moghaddam A (2018). Macrophage plasticity, polarization, and function in health and disease. Journal of cellular physiology.

[7] Jin Y-F, Han H-C, Berger J, Dai Q, Lindsey ML (2011). Combining experimental and mathematical modeling to reveal mechanisms of macrophage-dependent left ventricular remodeling. BMC systems biology.

[8] Wang Y (2012). Mathematical modeling and stability analysis of macrophage activation in left ventricular remodeling post-myocardial infarction. BMC genomics.

[9] Biswas, D., Roy, P. K., Li, X.-Z., Basir, F. A. & Pal, J. Role of macrophage in the disease dynamics of cutaneous Leishmaniasis: a delay induced mathematical study. *Communications in mathematical biology and neuroscience* **2016**, Article ID 4 (2016).

[10] Pertsovskaya I, Abad E, Domedel-Puig N, Garcia-Ojalvo J, Villoslada P (2013). Transient oscillatory dynamics of interferon beta signaling in macrophages. BMC systems biology.

[11] Moganti K (2017). Hyperglycemia induces mixed M1/M2 cytokine profile in primary human monocyte-derived macrophages. Immunobiology.

[12] Piccolo V (2017). Opposing macrophage polarization programs show extensive epigenomic and transcriptional cross-talk. Nature immunology.

[13] Yıldırım-Buharalıoğlu G, Bond M, Sala-Newby GB, Hindmarch CCT, Newby AC (2017). Regulation of epigenetic modifiers, including KDM6B, by interferon-γ and interleukin-4 in human macrophages. Frontiers in immunology.

[14] Wang, N., Liang, H. & Zen, K. Molecular Mechanisms That Influence the Macrophage M1–M2 Polarization Balance. *Frontiers in Immunology* **5**, 10.3389/fimmu.2014.00614 (2014). 10.3389/fimmu.2014.00614 PMC4246889 25506346

[15] Yan D, Wang H-W, Bowman RL, Joyce JA (2016). STAT3 and STAT6 Signaling Pathways Synergize to Promote Cathepsin Secretion from Macrophages via IRE1α Activation. Cell Rep.

[16] Snowden TJ, van der Graaf PH, Tindall MJ (2017). Methods of model reduction for large-scale biological systems: a survey of current methods and trends. Bulletin of mathematical biology.

[17] Kim J-R, Yoon Y, Cho K-H (2008). Coupled feedback loops form dynamic motifs of cellular networks. Biophysical journal.

[18] Tyson JJ, Novák B (2010). Functional motifs in biochemical reaction networks. Annual review of physical chemistry.

[19] Khallou-Laschet J (2010). Macrophage plasticity in experimental atherosclerosis. PloS one.

[20] Mantovani A, Biswas SK, Galdiero MR, Sica A, Locati M (2013). Macrophage plasticity and polarization in tissue repair and remodelling. The Journal of pathology.

[21] Wiggins, S. *Introduction To Applied Nonlinear Dynamical Systems And Chaos*. Vol. 2 (Springer Science & Business Media, 2003).

[22] Ermentrout, B. Xppaut. In *Computational Systems Neurobiology*, 519–531 (Springer, 2012).

[23] Martinez, F. O. & Gordon, S. The M1 and M2 paradigm of macrophage activation: time for reassessment. *F1000prime reports* **6** (2014). 10.12703/P6-13 PMC3944738 24669294

[24] Arora, S., Dev, K., Agarwal, B., Das, P. & Syed, M. A. Macrophages: their role, activation and polarization in pulmonary diseases. *Immunobiology* (2017). 10.1016/j.imbio.2017.11.001 PMC7114886 29146235

[25] Stöhr R, Federici M (2013). Insulin resistance and atherosclerosis: convergence between metabolic pathways and inflammatory nodes. Biochemical Journal.

[26] Maiti S, Dai W, Alaniz RC, Hahn J, Jayaraman A (2014). Mathematical modeling of pro-and anti-inflammatory signaling in macrophages. Processes.

[27] Wang Z, Butner JD, Kerketta R, Cristini V, Deisboeck TS (2015). Simulating cancer growth with multiscale agent-based modeling. in. Seminars in cancer biology.

[28] Wang M., Weiss M., Simonovic M., Haertinger G., Schrimpf S. P., Hengartner M. O., von Mering C. (2012). PaxDb, a Database of Protein Abundance Averages Across All Three Domains of Life. Molecular & Cellular Proteomics.

[29] Ross AE, Pompano RR (2018). Diffusion of cytokines in live lymph node tissue using microfluidic integrated optical imaging. Analytica chimica acta.

[30] Goodhill GJ (1997). Diffusion in axon guidance. European Journal of Neuroscience.

[31] Kihara T, Ito J, Miyake J (2013). Measurement of biomolecular diffusion in extracellular matrix condensed by fibroblasts using fluorescence correlation spectroscopy. PLoS One.

[32] Li C, Levin M, Kaplan DL (2016). Bioelectric modulation of macrophage polarization. Scientific reports.

[33] Godwin JW, Debuque R, Salimova E, Rosenthal NA (2017). Heart regeneration in the salamander relies on macrophage-mediated control of fibroblast activation and the extracellular landscape. NPJ Regenerative medicine.

[34] Nguyen-Chi M (2015). Identification of polarized macrophage subsets in zebrafish. eLife.

[35] Lee S (2011). Distinct Macrophage Phenotypes Contribute to Kidney Injury and Repair. Journal of the American Society of Nephrology.

[36] Das A (2015). Monocyte and Macrophage Plasticity in Tissue Repair and Regeneration. The American Journal of Pathology.

[37] Italiani P, Boraschi D (2014). From Monocytes to M1/M2 Macrophages: Phenotypical vs. Functional Differentiation. Frontiers in Immunology.

[38] Edin S, Wikberg ML, Rutegård J, Oldenborg P-A, Palmqvist R (2013). Phenotypic skewing of macrophages *in vitro* by secreted factors from colorectal cancer cells. PloS one.

[39] Bronte V, Murray PJ (2015). Understanding Local Macrophage Phenotypes In Disease: Modulating macrophage function to treat cancer. Nature Medicine.

[40] Sica A, Schioppa T, Mantovani A, Allavena P (2006). Tumour-associated macrophages are a distinct M2 polarised population promoting tumour progression: potential targets of anti-cancer therapy. European journal of cancer.

[41] Zhang L, Athale CA, Deisboeck TS (2007). Development of a three-dimensional multiscale agent-based tumor model: simulating gene-protein interaction profiles, cell phenotypes and multicellular patterns in brain cancer. Journal of theoretical biology.

[42] Troisi A, Wong V, Ratner MA (2005). An agent-based approach for modeling molecular self-organization. Proceedings of the National Academy of Sciences.

[43] Bailey AM, Thorne BC, Peirce SM (2007). Multi-cell agent-based simulation of the microvasculature to study the dynamics of circulating inflammatory cell trafficking. Annals of biomedical engineering.

[44] Walker DC, Hill G, Wood SM, Smallwood RH, Southgate J (2004). Agent-based computational modeling of wounded epithelial cell monolayers. IEEE transactions on nanobioscience.

[45] de Back W, Zhou JX, Brusch L (2013). On the role of lateral stabilization during early patterning in the pancreas. Journal of The Royal Society Interface.

[46] de Back W, Zimm R, Brusch L (2013). Transdifferentiation of pancreatic cells by loss of contact-mediated signaling. BMC systems biology.

[47] Ferrell JE (2009). Simple, realistic models of complex biological processes: positive feedback and bistability in a cell fate switch and a cell cycle oscillator. FEBS letters.

[48] Angeli D, Ferrell JE, Sontag ED (2004). Detection of multistability, bifurcations, and hysteresis in a large class of biological positive-feedback systems. Proceedings of the National Academy of Sciences.

[49] Pfeuty B, Kaneko K (2009). The combination of positive and negative feedback loops confers exquisite flexibility to biochemical switches. Physical biology.

[50] Tan C, Marguet P, You L (2009). Emergent bistability by a growth-modulating positive feedback circuit. Nature chemical biology.

[51] Couzin ID (2011). Uninformed individuals promote democratic consensus in animal groups. science.

[52] Leonard NE (2014). Multi-agent system dynamics: Bifurcation and behavior of animal groups. Annual Reviews in Control.

[53] Quinones M (2000). Preformed membrane-associated stores of interleukin (IL)-12 are a previously unrecognized source of bioactive IL-12 that is mobilized within minutes of contact with an intracellular parasite. Journal of Experimental Medicine.

[54] Navale AM, Paranjape AN (2013). Role of inflammation in development of diabetic complications and commonly used inflammatory markers with respect to diabetic complications. Int J Pharm Pharm Sci.

[55] Schindler H, Lutz MB, Röllinghoff M, Bogdan C (2001). The production of IFN-γ by IL-12/IL-18-activated macrophages requires STAT4 signaling and is inhibited by IL-4. The Journal of Immunology.

[56] Heller NM (2004). Interferon-γ inhibits STAT6 signal transduction and gene expression in human airway epithelial cells. American journal of respiratory cell and molecular biology.

[57] Andrews RP, Ericksen MB, Cunningham CM, Daines MO, Hershey GKK (2002). Analysis of the life cycle of Stat6 continuous cycling of Stat6 is required for IL-4 signaling. Journal of Biological Chemistry.

[58] Kim Hag Dong, Yu Su-Jin, Kim Hee Suk, Kim Yong-Jin, Choe Jeong Min, Park Yun Gyu, Kim Joon, Sohn Jeongwon (2013). Interleukin-4 Induces Senescence in Human Renal Carcinoma Cell Lines through STAT6 and p38 MAPK. Journal of Biological Chemistry.

[59] Sadzak I (2008). Recruitment of Stat1 to chromatin is required for interferon-induced serine phosphorylation of Stat1 transactivation domain. Proceedings of the National Academy of Sciences.

