# Supplementary material for: Agent-based modeling and bifurcation analysis reveal mechanisms of macrophage polarization and phenotype pattern distribution
Source: Sci Rep. 2019 Sep 4;9:12764. doi: 10.1038/s41598-019-48865-z (PMC6726649; doi:10.1038/s41598-019-48865-z)
Supplement: Supplementary file 1 — Supplementary Table S1 [file 41598_2019_48865_MOESM1_ESM.pdf]

Agent-based modeling and bifurcation analysis reveal mechanisms of macrophage polarization and phenotype pattern distribution

Authors: Niloofar Nickaeen[1], Jafar Ghaisari[1]*, Monika Heiner[2], Shiva Moein[4], Yousof Gheisari*[3, 4]

Addresses:

[1] Department of Electrical and Computer Engineering, Isfahan University of Technology, 84156-83111, Isfahan, Iran

[2] Computer Science Department, Brandenburg University of Technology, 03013 Cottbus-Senftenberg, Germany

[3] Department of Genetics and Molecular Biology, Isfahan University of Medical Sciences, Isfahan, 81746-73461, Iran

[4] Regenerative Medicine Research Center, Isfahan University of Medical Sciences, Isfahan 81746-73461, Iran

Emails:

Niloofar Nickaeen, n.nickaeen@ec.iut.ac.ir

Monika Heiner, monika.heiner@b-tu.de

Shiva Moein, shivamoein@gmail.com

Corresponding Authors:

1. Jafar Ghaisari, Department of Electrical and Computer Engineering, Isfahan University of Technology, Isfahan 84156-83111, Iran. Email: ghaisari@cc.iut.ac.ir.
2. Yousof Gheisari, Regenerative Medicine Research Center, Isfahan University of Medical Sciences, Isfahan 81746-73461, Iran. Email: ygheisari@med.mui.ac.ir.

Supplementary Table S1: Macrophage steady state status while it is simultaneously exposed to different ranges of IL4, LPS and IFNγ

#	IFNγ range	LPS Range	IL4 Range	Steady state status
1	IFNγ ≤ 0.14	LPS ≤ 0.37	IL4 ≤ 0.97	(Low, Low, High)
2	IFNγ ≥ 0.14	LPS ≤ 0.37	IL4 ≤ 0.97	(Low, High, Low)
3	IFNγ ≤ 0.14	0.37 ≤ LPS ≤ 1.64	IL4 ≤ 0.97	Multi-Stability
4	IFNγ ≥ 0.14	0.37 ≤ LPS ≤ 1.64	IL4 ≤ 0.97	(High, High, Low)
5	IFNγ ≤ 0.14	1.64 ≤ LPS	IL4 ≤ 0.97	(High, High, Low)
6	IFNγ ≥ 0.14	1.64 ≤ LPS	IL4 ≤ 0.97	(High, High, Low)
7	IFNγ ≤ 0.14	LPS ≤ 0.37	0.97 ≤IL4 ≤ 1.43	Multi-Stability
8	IFNγ ≥ 0.14	LPS ≤ 0.37	0.97 ≤IL4 ≤ 1.43	(Low, High, Med)
9	IFNγ ≤ 0.14	0.37 ≤ LPS ≤ 1.64	0.97 ≤IL4 ≤ 1.43	Multi-Stability
10	IFNγ ≥ 0.14	0.37 ≤ LPS ≤ 1.64	0.97 ≤IL4 ≤ 1.43	Multi-Stability
11	IFNγ ≤ 0.14	1.64 ≤ LPS	0.97 ≤IL4 ≤ 1.43	Multi-Stability
12	IFNγ ≥ 0.14	1.64 ≤ LPS	0.97 ≤IL4 ≤ 1.43	(High, High, Med)
13	IFNγ ≤ 0.14	LPS ≤ 0.37	1.43 ≤ IL4	(Low, Low, High)
14	IFNγ ≥ 0.14	LPS ≤ 0.37	1.43 ≤ IL4	(High, High, High)
15	IFNγ ≤ 0.14	0.37 ≤ LPS ≤ 1.64	1.43 ≤ IL4	(Low, Low, High)
16	IFNγ ≥ 0.14	0.37 ≤ LPS ≤ 1.64	1.43 ≤ IL4	Multi-Stability
17	IFNγ ≤ 0.14	1.64 ≤ LPS	1.43 ≤ IL4	Multi-Stability
18	IFNγ ≥ 0.14	1.64 ≤ LPS	1.43 ≤ IL4	(High, High, High)
